# A Case of Superfœtation

**Published:** 1881-01

**Authors:** D. A. Walden


### A CASE OF SUPERFŒTATION—BY DR. D. A. WALDEN.

A PRIMIPARA, aged twenty, married ten months, was taken with labor-pains April 18th, and next day was delivered of a full-term child. The patient did well until two days later. On that day, while using the vessel, there passed from her a complete unruptured sac, containing a male fœtus and placenta about four months old. The woman made a rapid recovery. She stated that she had menstruated for three or four months after she was aware of her pregnancy. There were no sighs of a double uterus. In a somewhat similar case, related by Playfair, menstruation was kept up during the whole period of pregnancy.—*Medical Record.*

